# Functional characterization of a panel of high-grade serous ovarian cancer cell lines as representative experimental models of the disease

**DOI:** 10.18632/oncotarget.9053

**Published:** 2016-04-27

**Authors:** James Haley, Sunil Tomar, Nicholas Pulliam, Sen Xiong, Susan M. Perkins, Adam R. Karpf, Sumegha Mitra, Kenneth P. Nephew, Anirban K. Mitra

**Affiliations:** ^1^ Medical Sciences Program, Indiana University School of Medicine, Bloomington, IN 47405, USA; ^2^ Department of Biostatistics, Indiana University, Indianapolis, IN 46202, USA; ^3^ Eppley Institute and Fred and Pamela Buffett Cancer Center, University of Nebraska Medical Center, Omaha, NE 68198, USA; ^4^ Department of Biochemistry and Molecular Biology, Indiana University School of Medicine, Indianapolis, IN 46202, USA; ^5^ Indiana University Melvin and Bren Simon Cancer Center, Indianapolis, IN 46202, USA; ^6^ Department of Cellular and Integrative Physiology, Indiana University School of Medicine, Indianapolis, IN 46202, USA; ^7^ Department of Medical and Molecular Genetics, Indiana University School of Medicine, Indianapolis, IN 46202, USA

**Keywords:** ovarian cancer, migration, invasion, proliferation, clonogenicity

## Abstract

Genomic analysis of ovarian cancer cell lines has revealed a panel that best represents the most common ovarian cancer subtype, high-grade serous ovarian cancer (HGSOC). However, these HGSOC-like cell lines have not been extensively applied by ovarian cancer researchers to date, and the most commonly used cell lines in the ovarian cancer field do not genetically resemble the major clinical type of the disease. For the HGSOC-like lines to serve as suitable models, they need to be characterized for common functional assays. To achieve that objective, we systematically studied a panel of HGSOC cells CAOV3, COV362, Kuramochi, OVCAR4, OVCAR5, OVCAR8, OVSAHO and SNU119 for migration, invasion, proliferation, clonogenicity, EMT phenotype and cisplatin resistance. They exhibited a range of efficacies and OVCAR5, OVCAR8 and Kuramochi were the most aggressive. SNU119 and OVSAHO cells demonstrated the lowest functional activities. Wide differences in expression of EMT markers were observed between cell lines. SNU119 were the most epithelial and OVCAR8 had the most mesenchymal phenotype. COV362 was the most resistant to cisplatin while CAOV3 was the most sensitive. Taken together, our systematic characterization represents a valuable resource to help guide the application of HGSOC cells by the cancer research community.

## INTRODUCTION

Ovarian cancer (OC) is the most lethal gynecological malignancy and is the 5th leading cause of cancer related deaths among women in the United States of America [1]. While many cancers have witnessed significant decrease in mortality in recent years due to advances in early detection and improved treatment options, OC death rates have remained relatively constant, with a 5-year survival rate under 30% for the past 40 years [2–4]. Discovery of effective therapeutics has been hampered by the lack of similarity between experimental models and real disease. Better models are needed if laboratory results are to be efficiently translated to patients [5, 6].

Until recently, a major issue with identifying good experimental models has been a lack of knowledge regarding the molecular and mutational profiles that are most characteristic of OC [6]. High-grade serous ovarian cancer (HGSOC) represents approximately 80% of OC [7] and contributes to two-thirds of all OC deaths [6], making it the most common as well as the most lethal subtype. Therefore, experimental models closely representing HGSOC are highly desirable.

The Cancer Genome Atlas Research Network (TCGA) study [8] revealed recurring genetic and molecular changes present in 489 clinically annotated stage-II–IV HGSOC tumors. Key recurring features of HGSOC included mutation of TP53 (~96% of tumors), loss of function or methylation in BRCA1/2, increased copy number variation, and statistically significant occurrence of somatic mutations in nine genes including NF1, RB1, and CDK12. The study also identifies a number of signaling pathways commonly altered in HGSOC, including the FOXM1 signaling pathway. A subsequent study [6] compared 47 cell line molecular profiles to the TCGA data and suggested that the most highly cited cell lines in research papers investigating HGSOC are not the cell lines most likely to actually represent HGSOC. Specifically, out of 47 cell lines used in the analysis, the two most highly cited - SKOV3 and A2780 - had some of the lowest suitability scores for HGSOC and were therefore ranked as “unlikely high grade serous.” A number of the cell lines listed as “likely high-grade serous” in this study have had this status confirmed in other studies using a variety of metrics [5, 7, 9]. Angleiso et al. reported a set of molecular features and biomarkers in a panel of ovarian cancer cells establishing their histotype [5]. A subsequent study by Beaufort et al. with a panel of ovarian cancer cell lines showed the cellular morphology correlated with specific biological and molecular characteristics [7]. Moreover, HGSOC cells like Kuramochi and OVSAHO were found to have significantly reduced capacity to form xenograft tumors as compared to SKOV3 cells [10].

The realization that the most commonly used cell lines in OC research are not representative of HGSOC will steadily lead to a shift towards using the more HGSOC-like cell lines. In order to be useful, these HGSOC cell lines need to be well characterized and should accurately represent the disease [4]. Since many of them are not well reported in literature, it is necessary to better understand their functional characteristics in order to widely apply them in OC research.

Recent studies have implicated EMT as a driver of OC metastasis [11–13]. An assessment of the basal expression of genes related to the EMT signature of different ovarian carcinoma cell lines would be key in understanding their metastatic potential. Additionally, since platinum is widely used as a first line of treatment in OC, knowledge of the response of these HGSOC cells to platinum would be very useful for researchers studying drug resistance or combination therapies.

We report the characterization of a panel of these HGSOC cell lines for commonly used *in vitro* functional assays, their sensitivity to cisplatin and their expression of epithelial and mesenchymal markers. The absence of published reports of such consolidated data hampers effective transition to the use of these HGSOC cell line models for ovarian cancer research. We believe that our data will be very beneficial to the field and will serve as a guide to optimize assay and treatment conditions for various mechanistic, drug development and screening studies. It will enable researchers to extensively use these to more accurately model OC.

## RESULTS

**Figure 1:**
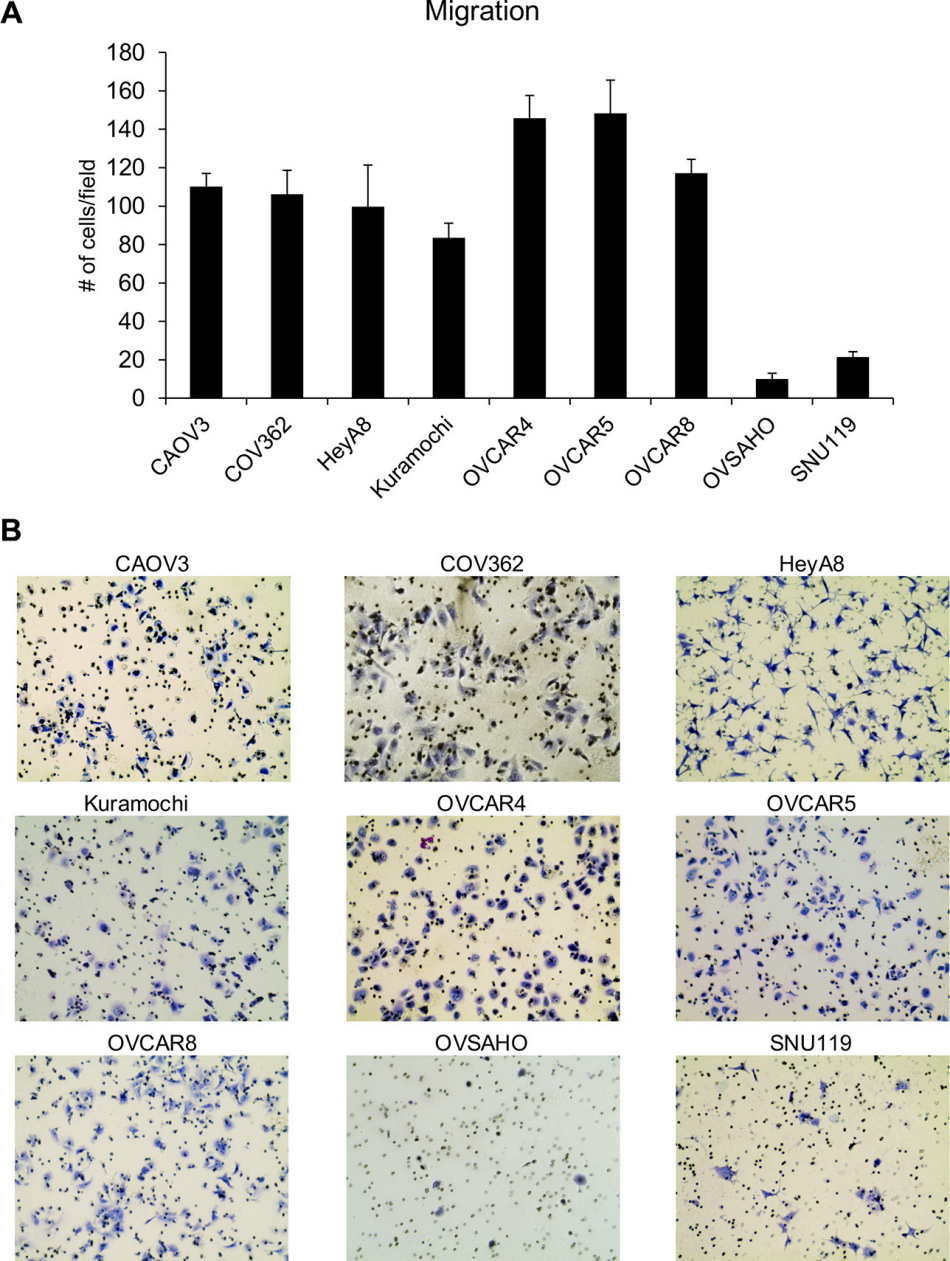
Comparison of the ability of the panel of HGSOC cells to migrate (**A**) Transwell migration assay was conducted using inserts with 8 μm pores and DMEM with 10% FBS as a chemoattractant. The number of migrated cells per field were imaged and counted (mean ± SD; 3 independent experiments). There were significant differences in the means across cell lines (*p* < 0.0001) as described in the results section. (**B**) Representative images of migrated cells for each cell line.

The ability of the HGSOC cell lines CAOV3, COV362, Kuramochi, OVCAR4, OVCAR5, OVCAR8, OVSAHO and SNU119 to migrate, invade, proliferate and form colonies was investigated. HeyA8 cells were also included in the set, as they have been very well characterized in all the four assays and serve as a control. Preliminary experiments were first conducted to identify the experimental conditions that were conducive to comparison of assay results between the cell lines. The final conditions used for migration, invasion, colony formation and proliferation assays for each cell line are listed in Table 1. The ability of cancer cells to respond to localized gradients of chemoattractants is considered crucial for metastasis [14]. Migration assays are extensively used *in vitro* to study the role of genes or effect of treatments on metastasis [15]. Transwell migration assays were conducted to compare the ability of the cell lines to move towards a chemoattractant (growth medium with 10% serum). The number of cells migrated per field was counted and data from the three independent experiments with each cell line is presented in Supplementary Figure 1 and the mean values for all cell lines are plotted together in Figure 1. OVCAR5 and OVCAR4 cells had the maximum number of migrated cells per field while OVSAHO and SNU119 had the least (Figure 1). There were significant differences in the means across cell lines (*p* < 0.0001). OVCAR5 and OVCAR4 were not different from each other but were different from all other cell lines. OVCAR8, CAOV3, COV362, and HeyA8 were not different from each other (with the exception of HeyA8 being different from OVCAR8), but were different from all other cell lines. Kuramochi was significantly different from all other cell lines. SNU119 and OVSAHO were not different from each other but were significantly different from all other cell lines. Since each cell line had a different propensity to migrate, the number of cells seeded per insert had to be varied between cell lines in order to obtain quantifiable migrated cell numbers. The migration was then normalized to the number of cells seeded and ranked accordingly (Table 2). Based on this, HeyA8 cells were found to have the greatest ability to migrate followed by OVCAR5 and OVCAR4 while OVSAHO and SNU119 remained the least migratory cells (Table 2). The cell sizes ranged between 15.78 μm to 20.31 μm (Supplementary Table 1).

**Table 1:** Functional assay conditions

Cell Line	Invasion/Migration Cell #	Migration Time	Invasion Time	Colony formation Cell #	Colony Formation Time	MTT Cell #	MTT Incubation Time	MTT Growth Time
CAOV3	200,000 cells	8 hrs	16 hrs	1,000 cells	22 days	10k, 5k, 2k, 1k	4 hrs	4 days
COV362	200,000 cells	8 hrs	16 hrs	1,000 cells	17 days	10k, 5k, 2k, 1k	4 hrs	4 days
HeyA8	50,000 cells	3 hrs	16 hrs	1,000 cells	7 days	10k, 5k, 2k, 1k	1.5 hrs	4 days
Kuramochi	200,000 cells	8 hrs	16 hrs	1,000 cells	14 days	10k, 5k, 2k, 1k	4 hrs	4 days
OVCAR4	200,000 cells	8 hrs	16 hrs	1,000 cells	14 days	10k, 5k, 2k, 1k	4 hrs	4 days
OVCAR5	200,000 cells	8 hrs	16 hrs	1,000 cells	14 days	10k, 5k, 2k, 1k	4 hrs	4 days
OVCAR8	200,000 cells	8 hrs	16 hrs	1,000 cells	14 days	10k, 5k, 2k, 1k	4 hrs	4 days
OVSAHO	500,000 cells	24 hrs	16 hrs	1,000 cells	28 days	10k, 5k, 2k, 1k	4 hrs	4 days
SNU119	200,000 cells	17 hrs	16 hrs	1,000 cells	14 days	10k, 5k, 2k, 1k	4 hrs	4 days

**Table 2:** Compilation of functional assay results

Cell Line	Migration			Invasion			Proliferation			Colony Formation		
	Mean (cell #)	SD	Rank	Mean (cell #)	SD	Rank	Mean (absorbance)	SD	Rank	Mean (colony #)	SD	Rank
CAOV3	110.2	6.8	5	10.2	1.9	7	1.3	0.2	4	148.2	17.5	8
COV362	106.2	12.5	6	11.4	5.0	6	1.1	0.1	6	164.7	15.8	7
HeyA8	99. 7	21.8	1	257.6	62.1	1	3.0	0.1	1	187.1	17.5	1
Kuramochi	83.5	7.6	7	35.1	2.2	4	0.9	0.1	7	238.9	16.7	3
OVCAR4	145.8	11.9	3	17.8	0.9	5	0.9	0.1	9	228.9	22.1	4
OVCAR5	148.3	17.3	2	268.7	19.1	2	1.5	0.2	2	199.2	15.4	5
OVCAR8	117.1	7.3	4	65.0	10.1	3	1.4	0.2	3	288.8	18.1	2
OVSAHO	10.0	3.0	9	0		8	1.2	0.3	5	43.4	2.0	9
SNU119	21.5	2.7	8	0		8	0.8	0.1	8	162.7	10.7	6

**Figure 2:**
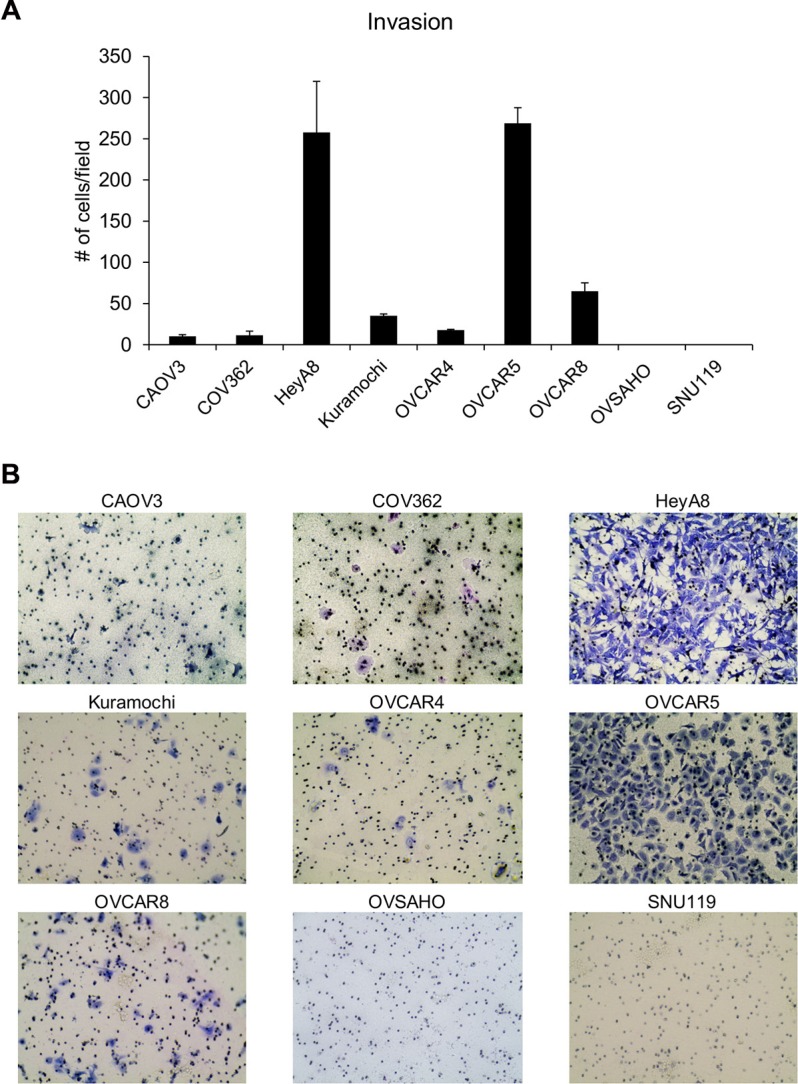
Evaluation of the ability of the panel of HGSOC cells to invade through matrigel (**A**) Transwell invasion assay was conducted using growth factor reduced matrigel coated inserts with 8 μm pores. DMEM with 10% FBS served as a chemoattractant. The number of invaded cells per field were imaged and counted (mean ± SD; 3 independent experiments). There were significant differences in the means across cell lines (*p* < 0.0001) as described in the results section. (**B**) Representative images of invaded cells for each cell line.

In addition to the ability to migrate towards chemoattractants, cancer cells need to have the ability to invade through the extracellular matrix surrounding cells and the basement membrane. Therefore, the ability of these cells to invade through matrigel was studied by using transwell invasion assay [16, 17]. The OC cells were allowed to invade through matrigel coated transwell inserts for 16 h. HeyA8 and OVCAR5 cells were found to be the most invasive while OVSAHO and SNU119 did not invade at all (Figure 2). There were significant differences in the means across cell lines (*p* < 0.0001). OVCAR5 and HeyA8 were not different from each other but were different from all other cell lines. OVCAR8 was different from all other cell lines, Kuramochi was not different from OVCAR4 but was different from all other cell lines. OVCAR4, COV362, and CAOV3 were not different but were different from all other cell lines. The independent experiments with each cell line are presented in Supplementary Figure 2. Normalizing the invasion to the number of cells seeded again resulted in HeyA8 cells as the most invasive followed by OVCAR5 (Table 2).

**Figure 3:**
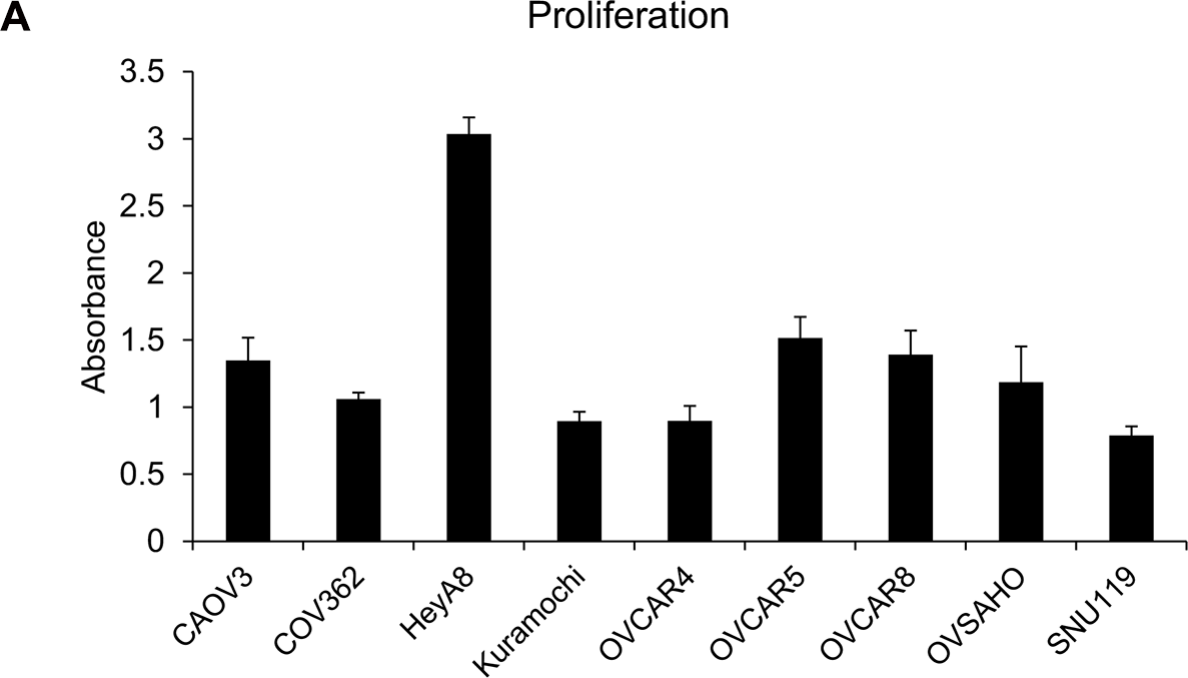
Capacity of the panel of HGSOC cells to proliferate *in vitro* The OC cells were seeded in 96-well plates (2000 cells/well) and allowed to grow for 4 days. Thereafter, their proliferation was measured using MTT assay and plotted to compare the growth rate of each cell line (mean ± SD; 3 independent experiments). There were significant differences in the means across cell lines (*p* < 0.0001) as described in the results section.

Having studied the ability of the OC cells to migrate and invade in response to chemoattractants, we proceeded to test their proliferation and clonogenicity. Both these traits are essential for tumor progression and are extensively used to study the effects of therapies, role of specific regulators or genes in OC [18, 19]. For proliferation, 1000, 2000, 5000 or 10,000 OC cells were seeded/well in 96-well plates and allowed to grow for 5 days. Proliferation was measured using the 3-(4, 5-dimethylthiazol-2-yl)-2, 5-diphenyltetrazolium bromide (MTT) assay, which is one of the most commonly used and economical assays to measure cell growth. The cell lines had varying rates of proliferation (Supplementary Figure 3) and only the experiment with 2000 cells seeded per well was found to be optimal for comparing the whole panel of cells (Figure 3). Once again, HeyA8 cells were found to be the most proliferative followed by OVCAR5 and OVCAR8. Kuramochi, OVCAR4 and SNU119 were found to be the least proliferative (Figure 3). There were significant differences in the means across cell lines (*p* < 0.0001). HeyA8 was different from all other cell lines. OVCAR5, OVCAR8 and CAOV3 were not different from each other but different from all other cell lines. COV362, Kuramochi, OVCAR4 and SNU119 were not different from each other (with the exception of COV362 being different from SNU119) but were different from all other cell lines.

**Figure 4:**
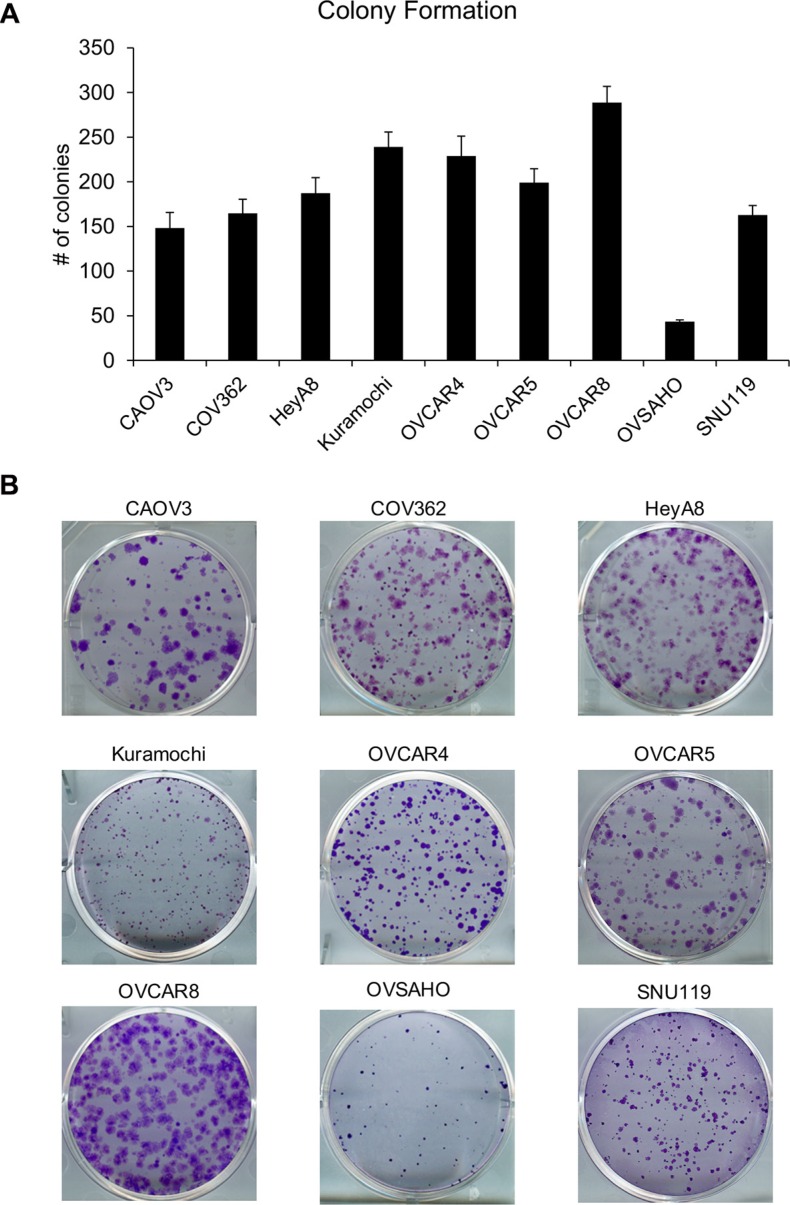
Clonogenic potential of the panel of HGSOC cells *in vitro* (**A**) The OC cells were seeded in 6-well plates (1000 cells/well) and allowed to grow to form visible colonies. Thereafter, the colonies were fixed, stained, imaged and counted to quantify the clonogenicity of these cell lines (mean ± SD; 3 independent experiments). There were significant differences in the means across cell lines (*p* < 0.0001) as described in the results section. (**B**) Representative images of colonies formed by each cell line.

To study the clonogenicity of the cell lines, colony formation assay was conducted by seeding 1000 cells/well of a 6-well plate and the resulting colonies were fixed, stained, imaged and counted. OVCAR8 formed the most colonies while OVSAHO formed the least (Figure 4). There were significant differences in the means across cell lines (*p* < 0.0001). Both OVCAR8 and OVSAHO were different from all other cell lines. Kuramochi and OVCAR4 were not different from each other but were different from all other cell lines. OVCAR5, HeyA8, COV362, SNU119, and CAOV3 were not different from each other (with the exceptions that OVCAR5 was different from COV362, SNU119, and CAOV3, while CAOV3 was also different from HeyA8). The time required for forming colonies ranged from 7 days for HeyA8 cells to 4 weeks for OVSAHO cells (Table 1). Therefore, in order to make an accurate comparison, the clonogenicity was normalized to the amount of time required to form visible colonies and the cell lines were ranked accordingly (Table 2). HeyA8 had the greatest clonogenicity followed by OVCAR8 while OVSAHO and CAOV3 cells had the lowest capacity to form colonies. The independent repeats for each cell line are plotted in Supplementary Figure 4.

**Figure 5:**
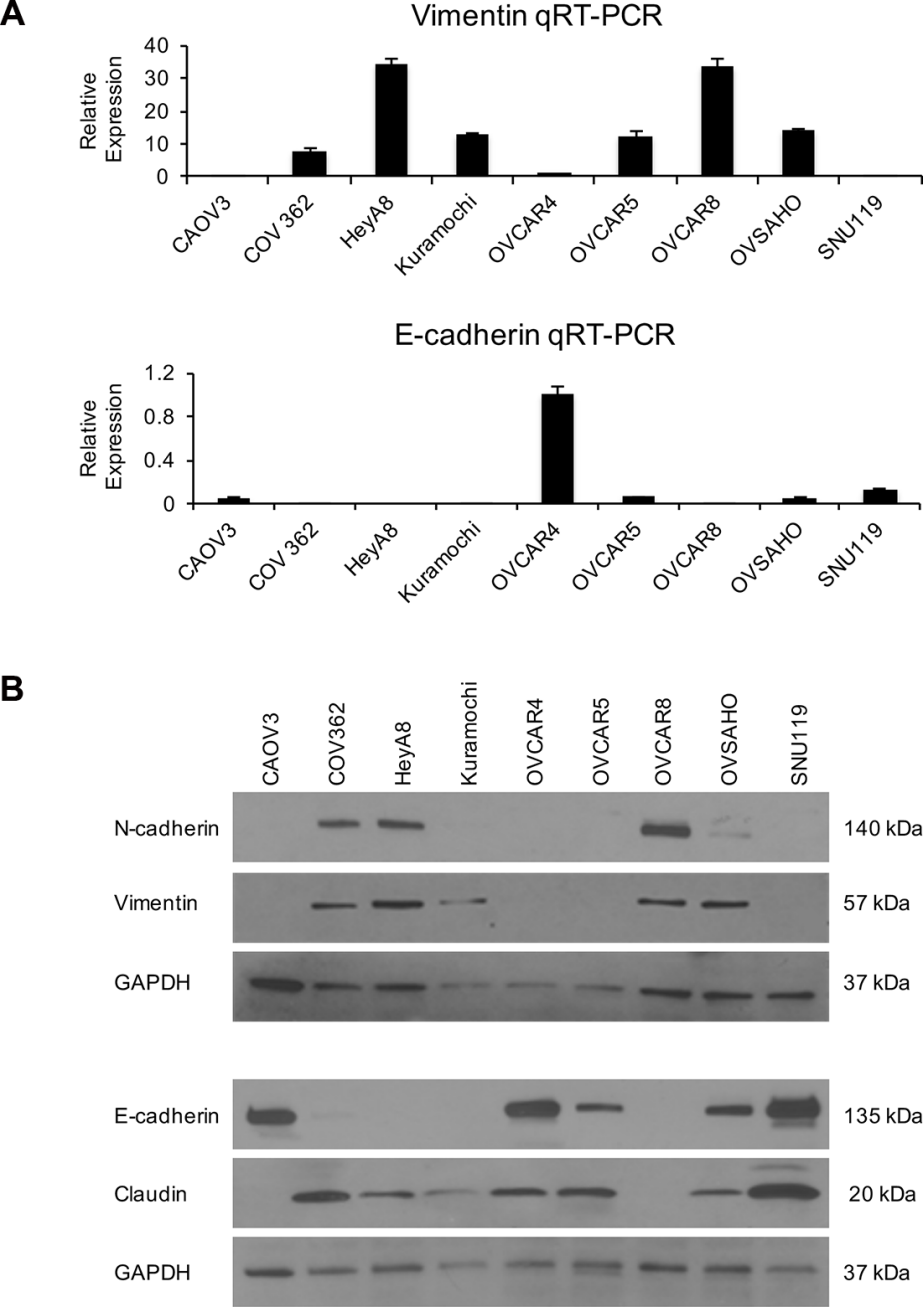
EMT status of the panel of HGSOC cells Total RNA was isolated from the OC cells and used for reverse transcription followed by qRT-PCR using TaqMan gene expression assays (**A**) for vimentin and E-cadherin. Relative quantification was done using GAPDH as an internal control (mean ± SD; 3 independent experiments). (**B**) Western blot analysis of N-cadherin, vimentin, E-cadherin and claudin expression in the HGSOC cell lines using GAPDH as a loading control. Representative images of 3 independent experiments are shown.

A key feature of cancer progression is EMT. Therefore, we characterized the HGSOC cell lines for their expression of the epithelial markers E-cadherin and claudin and mesenchymal markers vimentin and N-cadherin. Total RNA was obtained from the cell lines and qRT-PCR was performed for vimentin and E-cadherin. Vimentin expression levels were the maximum in HeyA8 and OVCAR8 cells and were the lowest in SNU119 and CAOV3 cells (Figure 5A). OVCAR4 had the highest E-cadherin expression and HeyA8 and OVCAR8 had the least (Figure 5A). Western blotting was also done to study the expression of all the 4 markers. The protein expressions of both N-cadherin and vimentin were highest in OVCAR8 and HeyA8 cells while SNU119 had the maximum expression of E-cadherin and claudin (Figure 5B). The HGSOC cells having high levels of E-cadherin had lower expression of vimentin and vice versa. Interestingly, CAOV3 had minimal expression of the mesenchymal markers and high expression of E-cadherin but did not express claudin.

**Figure 6:**
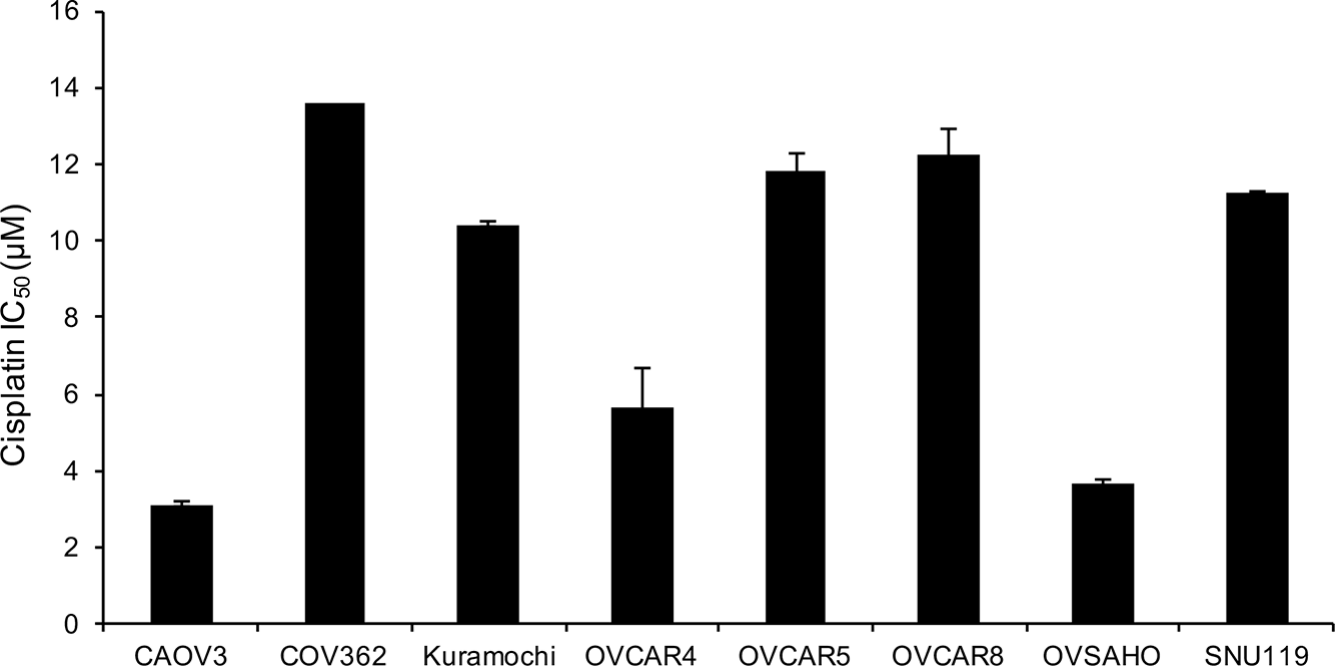
Cisplatin resistance of the panel of HGSOC cells The IC_50_ values for cispatin were determined in the cell lines by treating them with increasing doses of cisplatin for 24 h. This was followed by a 3-day recovery and then MTT assay was conducted to measure viability. IC_50_ was determined using Graph Pad (mean ± SD; 3 independent experiments).

Cell lines are the most commonly used models to test new drugs, combination therapies and chemoresistance [18, 20, 21]. Since platinum based therapies are the standard of care for OC patients, we proceeded to establish the IC_50_ dose for cisplatin in these HGSOC cell lines. Having this information would be useful for researchers using these cells for chemoresistance experiments and for studying combination of different drugs with platinum. COV362 had the highest IC_50_ dose for cisplatin (13.57 μM) and CAOV3 was the most sensitive (IC_50_ dose of 3.14 μM) to cisplatin (Figure 6).

## DISCUSSION

To conduct *in vitro* experiments to study OC, it is essential to use cell lines that accurately represent the disease. The TCGA study was an important step towards genetic characterization of OC [8] and subsequent reports revealed that the most commonly used cell lines to model OC *in vitro* like SKOV3 and A2780 are not the best models for HGSOC [6]. While recent reports have provided important insights into the genetic characteristics of the cell lines that were found to be the best representatives of HGSOC [5–7], very limited literature is available about their functional characteristics. In order to effectively use these cellular models extensively for various aspects of OC research, it is essential to study their proliferation, clonogenicity, motility, invasiveness, drug resistance etc. *in vitro* and tumorigenicity *in vivo*. We have recently reported the tumorigenic ability of a panel of these HGSOC cell lines in mouse xenografts [22] and have now extended the study to characterization of their functional abilities *in vitro*, their EMT status as well as their resistance to cisplatin.

The present study is the first to systematically compare the *in vitro* functional characteristics of a set of eight HGSOC cell lines. The HGSOC cell lines exhibited a wide range of functional activities for each of the four assays tested as well as their EMT phenotype and cisplatin resistance. Therefore, they accurately represent the variability that is characteristic of the disease. It was interesting to note that some of the cellular models that most closely resemble HGSOC genetically (OVSAHO, SNU119 and Kuramochi) were much less potent in the functional assays. These *in vitro* functional assay results mirrored their ability to form mouse xenograft tumors reported previously [22]. HeyA8 cells, which were used as a reference for all the functional assays, were found to be much more aggressive compared to the HGSOC cells. It is important to note that HeyA8 cells have been listed as ‘unlikely high-grade serous’ [6]. Similarly SKOV3 and A2780 are also reported to be more aggressive [17, 18, 23]. Therefore, we can conclude that the cell lines that genetically resemble HGSOC are less potent in the functional assays. This would also agree with the fact that the growth rate of HGSOC tumors in patients or in patient-derived xenograft models is much slower than that observed for HeyA8, SKOV3.ip1 and A2780 xenografts [16, 18, 23–25]. These non-HGSOC cells have various combinations of mutations in the PI3-kinase, BRAF, ARID1A pathways which might help them perform better in the functional assays than the HGSOC cells [6]. This should be taken into consideration when planning experiments to test the effects of specific factors on tumor function.

There was no clear correlation between genetic resemblances to HGSOC and the EMT phenotype. All the cell line models were found to be relatively sensitive to cisplatin, which agreed with the previous report on COV362 and CAOV3 [7]. Since most HGSOC patients respond well initially to platinum therapy, these cell lines would serve as suitable models for studying response to platinum treatment and development of chemoresistance.

In conclusion, these HGSOC cell line models can be used as better representatives of the most common form of the disease. However, there are significant variations in their functional abilities, their EMT status as well as their sensitivity to cisplatin. Therefore, it is imperative that the assay conditions be adapted to the fact that some of these are less aggressive in the common functional assays tested compared to the OC cell lines that have been predominantly used. The standardized conditions reported herein would be useful as an appropriate starting point.

## MATERIALS AND METHODS

### Reagents

Dulbecco's Modified Eagle's Medium (DMEM) (Catalogue # 10-013-CV), MEM vitamins (Catalogue # 25-020-CI), MEM nonessential amino acids (Catalogue # 25-025-CI), Penicillin-Streptomycin (Catalogue # 30-002-CI) and trypsin (Catalogue # 25-053-CI) were purchased from Corning. FBS was purchased from Gibco (LifeTechnologies, catalogue # 16000-044). Giemsa stain (Catalogue # GS500) and crystal violet (Catalogue # C0775) were obtained from Sigma Aldrich. Thiazolyl blue tetrazolium bromide was from Acros Organics (Catalogue # 298-93-1) and 4% paraformaldehyde solution was purchased from Fisher Scientific (NC9245948).

### Cell lines

All cell lines were grown in DMEM with 10% FBS along with 1% MEM vitamins, MEM nonessential amino acids and Penicillin-Streptomycin. CAOV3, HeyA8, OVCAR5 and OVCAR8 were obtained from Ernst Lengyel, University of Chicago and OVCAR4 from Joanna Burdette, University of Illinois at Chicago. OVSAHO was obtained from the Japanese Collection of Research Bioresources and SNU119 was from the Korean Cell Line Bank. COV362 (Sigma), and Kuramochi (Japanese Collection of Research Bioresources). All cell lines used were genetically validated and also confirmed to be mycoplasma free using respective services from Idexx BioResearch (Columbia, MO). The genetic validation was done using the CellCheck 16 (16 Marker STR Profile and Inter-species Contamination Test) and mycoplasma testing was done using Stat-Myco.

### Migration

Transwell migration assays were conducted using 8 μm pore size inserts (BD, Falcon Cat#353097). OC cells were trypsinized, recovered for 30 min at 37°C in the CO_2_ incubator (5% CO_2_) and then seeded in the upper chamber in 500 μl of serum free DMEM and allowed to migrate at 37°C. The numbers of cells seeded per insert and migration times are included in Table 1. DMEM with 10% FBS was used as a chemoattractant in the lower chamber. At the endpoint, cells were fixed in 4% paraformaldehyde for 30 min at room temperature, stained with Giemsa for 3 h followed by rinsing with distilled water and thorough wiping of the inside surface of the transwell membrane to remove any cells that have not migrated. The inserts were air-dried and imaged (5 fields/insert) using an EVOS FL Auto microscope (Life Technologies). Since the number of cells and the migration times varied between cell lines, the results were normalized to the number of cells seeded and the time of migration. Since the majority of cells were seeded at 200,000 cells/insert the cell number adjustment factor (N) was determined as follows:

N = 200,000/number of cells seeded for the cell line

Similarly, the baseline migration time was set at 8 h and the migration time adjustment factor (T) was calculated as follows:

T = 8/migration time for the cell line

The final adjusted cell count took into account the cell number adjustment factor (N) and the migration time adjustment factor (T) and was determined by:

Adjusted cell count = average number of cells per field × N × T

### Invasion

Cellular invasion through the extracellular matrix proteins was assayed using growth factor reduced matrigel coated transwell inserts (8 μm pore size, BD Falcon Cat# 354483) in 24-well plates. OC cells were trypsinized, recovered for 30 min at 37°C in the CO_2_ incubator and then seeded in the transwell insert in 500 μl serum-free DMEM. The number of cells seeded per insert are included in Table 1. DMEM with 10% FBS served as a chemoattractant in the lower chamber. Cells were allowed to invade for 16 h and then were fixed with 4% paraformaldehyde. The OC cells that had invaded were stained with Giemsa for 3 h followed by rinsing with distilled water and thorough wiping of the inside surface of the transwell membrane to remove any cells that have not migrated. The inserts were air-dried and imaged (5 fields/insert) using an EVOS FL Auto microscope (Life Technologies). The number of cells seeded per insert varied between cell lines but the invasion time was constant. Therefore, the normalization method used for migration was modified to exclude the time factor as follows:

Adjusted cell count = average number of cells per field × N

Where N = 200,000/number of cells seeded for the cell line.

### Proliferation

OC cells were seeded in 96-well plates in 8 replicates and allowed to grow for 5 days. To identify the optimal number of cells for comparison between cell lines, 1000, 2000, 5000 and 10000 cells of each cell line were seeded per well. On the fourth day MTT (3-(4, 5-dimethylthiazol-2-yl)-2,5-diphenyltetrazolium bromide) Assay was conducted to measure proliferation of the OC cells. 20 μl of MTT solution (5 mg/ml in sterile PBS) was added per well, mixed and incubated at 37°C. The incubation times for the OC cell lines are listed in Table 1. Thereafter, the colored product (formazan) was solubilized in DMSO and the absorbance measured at 560 nm and adjusted for background absorbance at 670 nm using a SynergyH1 plate reader (BioTek).

### Colony formation

OC cells were seeded in 6-well plates (1000 cells/well, 6 replicates) and allowed to form colonies. Medium was changed every fifth day. Once visible colonies were formed (cell line specific colony formation times are listed in Table 1), they were fixed with 4% paraformaldehyde and stained with 0.005% crystal violet and were imaged using a Syngene G:Box imaging system and the number of colonies/well were counted. The time required to form visible colonies varied between cell lines and therefore the colony counts were normalized for the time of growth. The baseline colony formation time was set at 14 days and the time adjustment factor (T) was calculated as follows:

T = 14/colony formation time for the cell line

Thereafter, the final adjusted colony count was determined as follows:

Adjusted colony count = Average number of colonies × T

### Cisplatin IC_50_

OC cells were plated at 2000 cells per well in a 96-well plate and 48 h after plating, cells were treated with increasing concentrations of cisplatin for 24 h. Thereafter, medium was changed and cells were allowed to recover for 72 h followed by MTT assay. IC_50_ was determined using Graph Pad Prism.

### Real time polymerase chain reaction

Quantitative real time polymerase chain (qRT-PCR) reaction was done for E-cadherin and vimentin using TaqMan gene expression assays as described previously [15]. Briefly, RNA was isolated from the cells using miRNeasy Kit (Qiagen Catalogue # 217004) and 1 μg RNA was used for reverse transcription using Applied Biosystems High Capacity Reverse Transcription Kit (Catalogue # 4368813). The relative E-cadherin and vimentin mRNA expression levels were determined using TaqMan gene expression assays (Applied Biosystems catalogue # Hs01023894_m1 and Hs00185584_m1 respectively). GAPDH was used as an internal control. qPCR was done using Roche LightCycler 96 system and the RT was done using a Veriti 96-well thermal cycler (Applied Biosystems).

### Western blotting

Western blotting was done as described previously [15]. Briefly, cells were lysed in ice-cold RIPA buffer and proteins were resolved using 4–20% gradient sodium dodecyl sulfate polyacrylamide gel electrophoresis followed by transfer to nitrocellulose membrane. Anti N-cadherin, E-cadherin, vimentin and claudin antibodies (Cell Signaling EMT Antibody Sampler Kit, Catalogue # 9782S) were used to probe for the respective proteins and detected using a horseradish peroxidase-linked anti-rabbit IgG secondary antibody (Cell Signaling, Cat#7074). Membranes were stripped and re-probed with HRP linked anti-GAPDH antibody (Sigma catalogue # G9295).

### Cell size measurements

Cell diameter was analyzed using the Z2 coulter Particle Count and Size Analyzer (Beckman Coulter, Miami, FL) using a 100 μm aperture size. Briefly, cells were diluted 1:10 in diluent (Isoton II) and 0.5 ml was loaded into the counter. All particles over 12 um were counted and measured. Between sample readings, the counter was flushed with the diluent.

### Statistics

ANOVA was used to compare mean migration, invasion, proliferation, and colony formation by cell line. The *p*-value from the overall *F*-test is reported. Tukey's method was used for pair-wise comparisons. The overall error rate per outcome was controlled at 5%.

## SUPPLEMENTARY MATERIALS FIGURES AND TABLE


