# Supplementary material for: Functional characterization of a panel of high-grade serous ovarian cancer cell lines as representative experimental models of the disease
Source: Oncotarget. 2016 Apr 27;7(22):32810–20. doi: 10.18632/oncotarget.9053 (PMC5078053; doi:10.18632/oncotarget.9053)
Supplement: Supplementary file 1 [file oncotarget-07-32810-s001.pdf]

Functional characterization of a panel of high-grade serous ovarian cancer cell lines as representative experimental models of the disease

Supplementary Materials

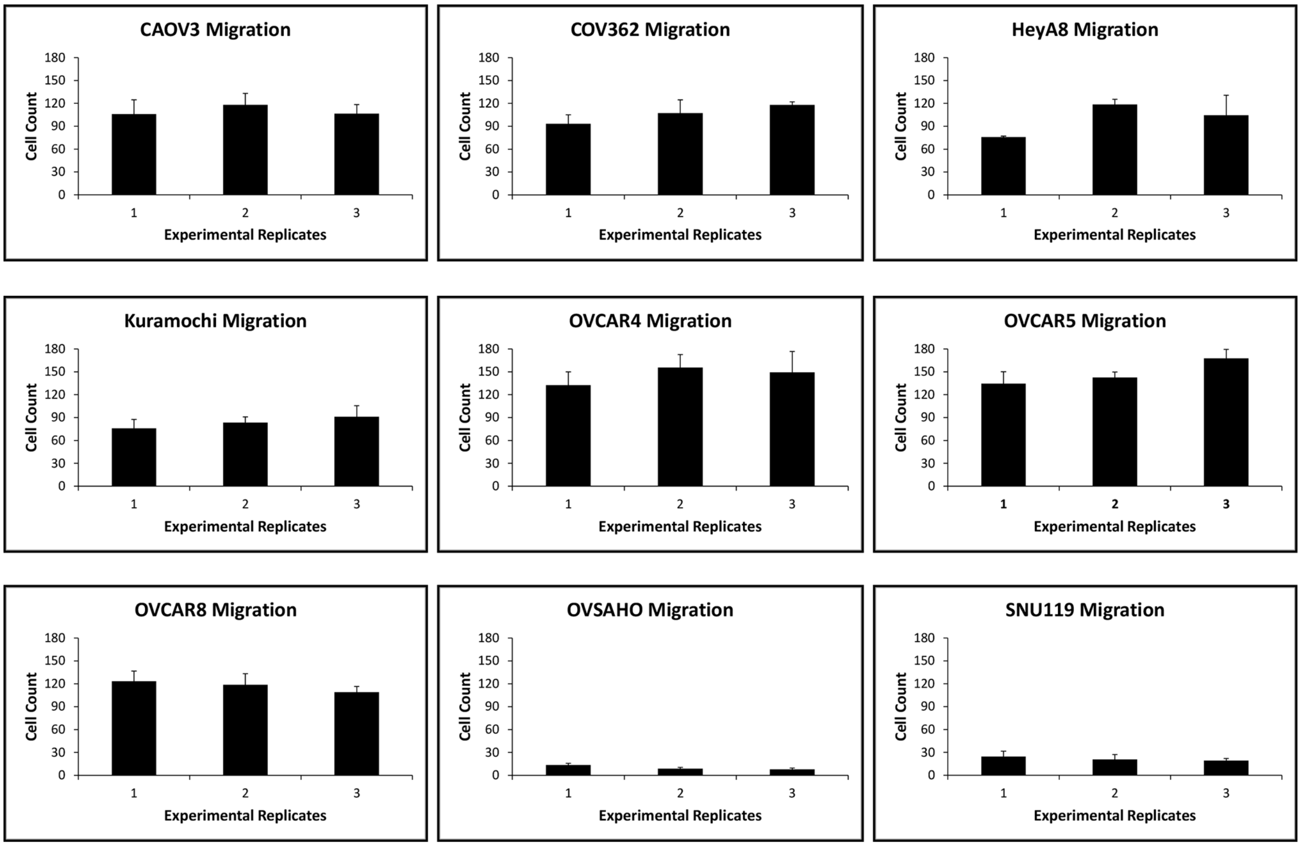

Supplementary Figure S1: A compilation of the migration assays for individual cell lines. The cells were seeded in transwell inserts with 8 µm pores and allowed to migrate towards DMEM with 10% FBS (chemoattractant). The migrated cells were fixed, stained with Giemsa and imaged. The plots represent 3 independent experiments each done in duplicate.

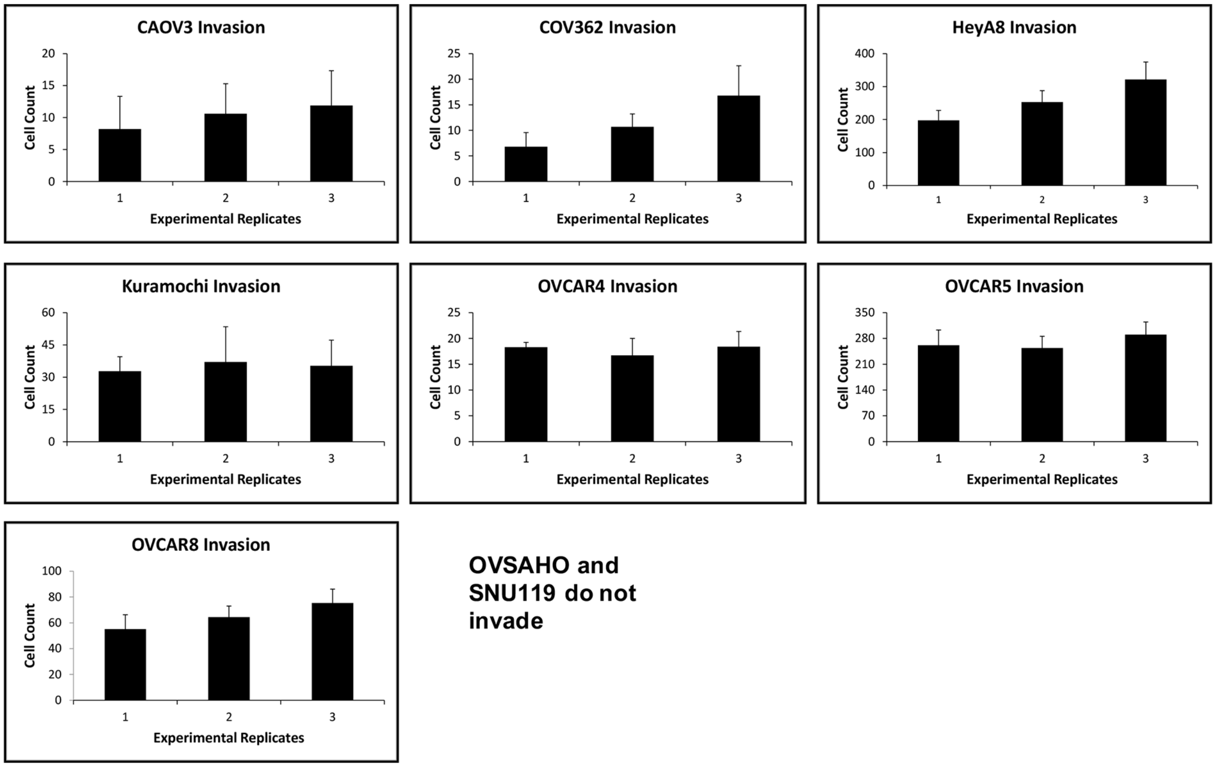

Supplementary Figure S2: A compilation of the invasion assays for individual cell lines. The cells were seeded in growth factor reduced matrigel coated transwell inserts with 8μm pores and allowed to invade towards DMEM with 10% FBS (chemoattractant). The invaded cells were fixed, stained with Giemsa and imaged. The plots represent 3 independent experiments each done in duplicate.

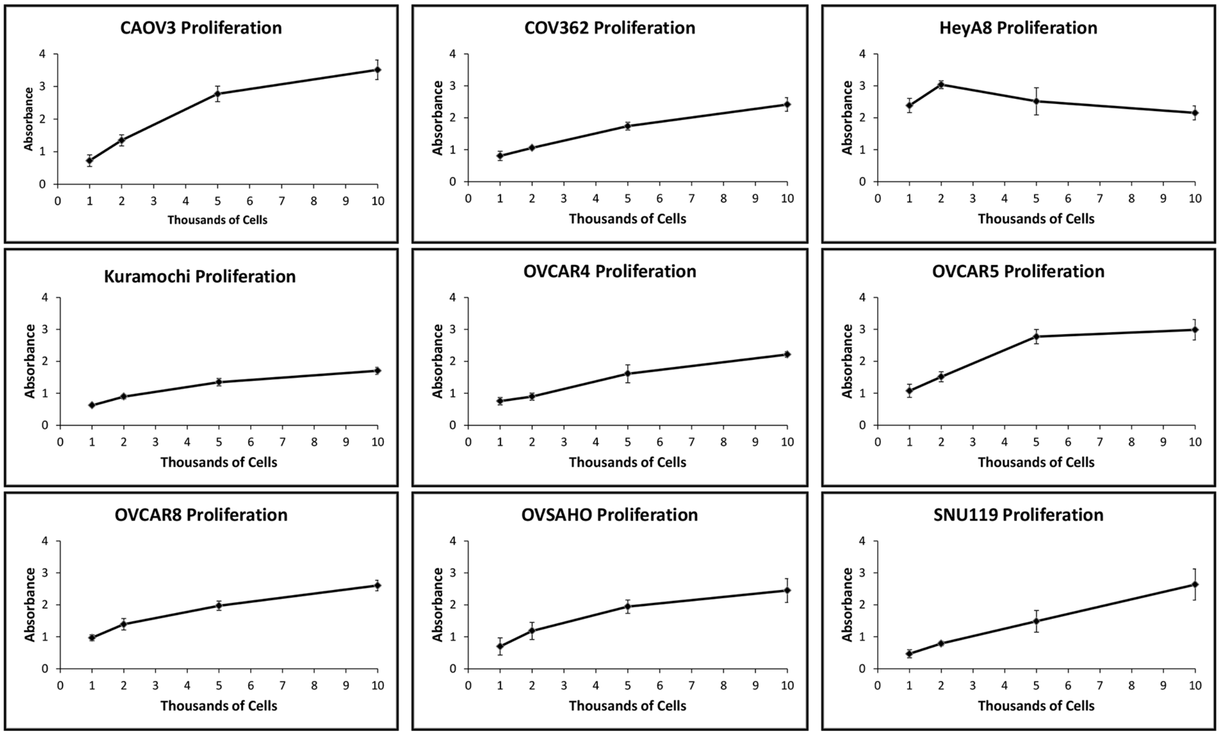

Supplementary Figure S3: A compilation of the proliferation assays for individual cell lines. The cells were seeded in 96-well plates (1000, 2000, 5000 or 10000 cells/well) in 8 replicates. The cells were allowed to grow for 4 days and the proliferation was measured using MTT assay. The plots represent data from 3 independent experiments.

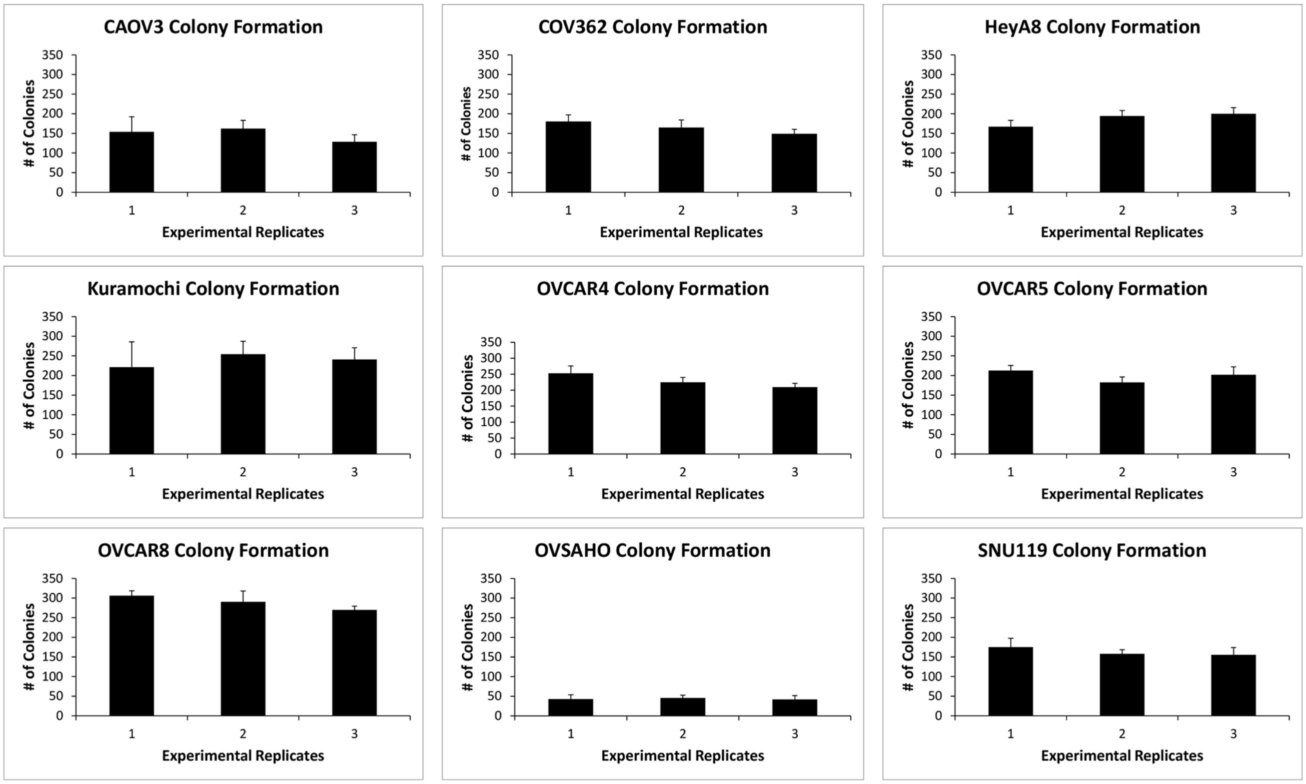

Supplementary Figure S4: A compilation of the colony formation assays for individual cell lines. The cells were seeded in 6-well plates (1000 cells/well) in 6 replicates. The cells were allowed to grow and form visible colonies which were fixed, stained with crystal violet, imaged and counted. The plots represent data from 3 independent experiments.

Supplementary Table S1: Cell diameters

Cell Line	Cell Diameter (um)
CAOV3	18.38
COV362	20.31
HeyA8	15.78
Kuramochi	17.63
OVCAR4	18.01
OVCAR5	15.81
OVCAR8	16.56
OVSAHO	16.81
SNU119	16.50
